# Multimodal behavioral treatment of migraine: An Internet-administered, randomized, controlled trial

**DOI:** 10.3109/03009734.2011.575963

**Published:** 2011-06-29

**Authors:** Kerstin Hedborg, Carin Muhr

**Affiliations:** ^1^Faculty of Health and Occupational Studies, Department of Health and Caring Sciences, University of Gävle, Sweden; ^2^Department of Medical Sciences, Neurology, Uppsala University, Sweden; ^3^Department of Medical Sciences, Uppsala University, Sweden

**Keywords:** Cognitive behavioral treatment, hand massage, Internet, migraine, multimodal, stress

## Abstract

**Introduction:**

Multimodal approaches in behavioral treatment have gained recent interest, with proven efficacy for migraine. The utility of the Internet has been demonstrated for behavioral treatment of headache disorders, but not specifically for migraine. The aim of the study was to develop and evaluate an Internet-based multimodal behavior treatment (MBT) program for migraine and to test hand massage treatment as an adjunct.

**Methods:**

Eighty-three adults, 58 women and 25 men, with at least two migraine attacks a month were recruited via advertisements. An MBT program aiming at improvements in life-style and stress coping was developed for this study and, together with a diary, adapted for use over the Internet. Participants were randomized to MBT with and without hand massage and to a control group, and were followed for 11 months. Questionnaires addressing issues of quality of life (PQ23) and depressive symptoms (MADRS-S) were used.

**Results:**

A 50%, or greater, reduction in migraine frequency was found in 40% and 42% of participants of the two groups receiving MBT (with and without hand massage, respectively), who statistically were significantly more improved than participants in the control group. No effect of hand massage was detected, and gender did not show any independent contribution to the effect in a multivariate analysis.

**Conclusions:**

MBT administered over the Internet appears feasible and effective in the treatment of migraine, but no effect of hand massage was found. For increased knowledge on long-term effects and the modes of action of the present MBT program, further studies are needed.

## Introduction

Multimodal behavioral modification programs utilize a holistic perspective (1) and aim at achieving broad-based cognitive and life-style changes. They have demonstrated efficacy in the treatment of pain disorders (2–6), including headache (3,5,6), and have been the subject of increased interest during recent years (1).With regard to migraine, using a variety of behavior therapies, alone or mixed, such as cognitive therapies, life-style modifications, bio-feedback training, and relaxation training, has shown efficacy (7–10), and two studies have used a multimodal approach (9,10).

In recent decades, the arsenal for pharmacological intervention in migraine has broadened, and the prospect of rapid symptom alleviation has improved considerably, but pharmacological treatment of migraine is still far from optimal (11). Also, migraine medications can have adverse side-effects or sometimes be ineffective (11,12). Therefore, and because migraine is highly conditional on stress factors (13), it would be beneficial to find non-pharmacological forms of treatment that are focused on relevant psycho-social factors. Multimodal behavior treatment is therefore of clear interest in migraine treatment. Furthermore, pharmacological and behavioral oriented treatments have shown additive effects in relation to migraine (12,14–16).

The role of massage in pain and headache treatment is controversial (1,8,17,18). Positive effects, including a decreased headache frequency and improved sleep quality, have been shown (19,20) presumably through increased muscular relaxation and decreased bodily symptoms of stress (21).

The Internet offers possibilities to increase the availability of different kinds of treatment programs at a low cost, and the efficacy of such web-based health care programs has been demonstrated for a variety of conditions (22,23). This particular advantage of Internet-based programs is valuable in relation to migraine, as many migraineurs have poor access to specialized health care (24–26). Only a few such Internet-based programs have addressed headache, and these have included migraine as one of the headache types; no such program has addressed migraine exclusively. We have thus identified three Internet-based studies on behavioral treatment of headache (27–29), all of which included mixed headache populations, with participants who have migraine and/or tension-type headache. Two of these Internet-based studies were from the same research group (27,29), and the same 6-week program for relaxation and cognitive behavior therapy was used in both studies. In the first study, consisting of 102 participants, significant improvements in headache frequencies were obtained. However, a high attrition rate of 56% made the result somewhat difficult to evaluate. In the second study, telephone contacts were added in an attempt to decrease the number of drop-outs. This study, too, revealed significant improvements in headache, but no statistically significant decrease in drop-outs was seen. A further, purely Internet-based study on chronic headache included one group with migraine/mixed headache and one with solely tension-type headache (28). However, because the results from the headache groups were merged, the specific effect of the migraine/mixed headache treatment was not known. The total attrition rate was 38% out of an initial treatment group of 139 participants. These three studies demonstrate that the Internet is useful in the administration of programs for relaxation and cognitive treatment of headache, although web-based programs seem associated with a high attrition rate.

Thus, to date, Internet-based behavioral treatment programs for headache have not utilized a comprehensive multimodal approach, and have not specifically addressed migraine (1,6,27–29). The primary aim of the present study was therefore to develop and evaluate such a migraine-specific Internet-administered multimodal behavioral treatment (MBT) program. The intention of this program was to improve participants' knowledge of stress physiology and awareness of factors of importance for daily stress and of the beneficial effects of physical activity, diet, as well as cognitive aspects with regard to thought patterns, handling of emotions, and attitudes. Hand massage was also randomly tested as an additional treatment modality.

We hypothesized that both the Internet-administered MBT program and the hand massage would be effective in decreasing the frequency of migraine. Therefore our primary outcome measure was reduction of migraine frequency by 50% or more. Secondary outcome measures were: physical activity, scores for depression and well-being, and participants' evaluations of the MBT program and of the hand massage.

## Material and methods

### Study population

The study population was recruited by approaching participants in a previous descriptive study on migraine. These 150 individuals had been recruited via advertisements in the local daily newspaper, asking people to participate in a 2-hour interview concerning their migraine. The inclusion criteria used for both studies were: fulfillment of migraine criteria according to the International Classification of Headache Disorders (30) and at least two migraine attacks monthly, as reported at the time of inclusion. At this initial session, a general medical and a specific headache history were recorded, followed by a general and neurological physical examination by one of the authors, a specialist in neurology. A battery of computer-based questionnaires was also answered by the participants. Eighty-three individuals, all adults, 58 women and 25 men, agreed to participate in the present study—all of whom were included after giving their verbal informed consent (ethical approval no.: Dnr Ups 03-189 Regional Ethical Review Board, Uppsala, Sweden). All participants were Swedish speaking, and the great majority of them lived in the city of Uppsala or its vicinity. Other than traveling costs, no financial compensation was offered.

### Web tool for migraine diary and for the MBT program

The authors, together with a professional advisor in stress management, developed an MBT program for migraine adapted for scientific purposes. A web-based tool was developed with support from web-designers; it contained the treatment program and a diary. Participants accessed the tool via a personal log-in. The diary was filled in day-by-day. In order to register on the next day, the program required that data for the previous day first be completed. A paper version of the diary could be used instead if participants did not have immediate access to the Internet. They were asked to transfer the data from the paper version to the web-based diary within one week; otherwise an automatic e-mail reminder would appear informing them that the missing data were necessary for continued diary recording. Via the web tool, participants were also asked to answer two questionnaires regarding depression and quality of life, repeated at 3–5-month intervals. The items recorded in the diary that were relevant for the present study were: *migraine frequency*, defined as number of days with migraine; *physical activity*, recorded as the number of days with physical activity here defined as brisk walks, cycling, and all forms of more extensive physical activities lasting 30 minutes or more; and intake of *migraine medication*. The MBT program was intended to increase participants' awareness of essential factors in everyday life that might have an impact on their migraine. This training program consisted of 53 text pages divided into the following topics: stress physiology, physical activity, diet, thought patterns, handling of emotions, and attitudes (toward oneself and others). A summary of the content of these topics and associated recommendations for behavioral change are shown in Table I. Physical activity lasting 30 minutes or more was recorded in the diary. Participants were told to use migraine drugs at their own discretion, independently of the study. The respective topics of the program included a reference list for eventual further reading. Print-outs were made possible by attached PDF files. The Swedish language was used throughout in the web tool.

**Table I.** Summary of content of the multimodal behavior treatment program.

Topic	Background information	Recommended behavioral change
Stress physiology	Concept of stress as a specific syndrome of bodily and mental reactions to unspecific strain; importance of cognitive processes for perceived stress; difference between acute and chronic stress; role of stress in migraine; importance of relaxation and sleep habits to mitigate stress reactions	(i) Identify own symptoms of stress and own protective ‘peace-and-rest’ reactions against stress (ii) Identify personal stressors in daily life, and analyze how they can be diminished (iii) Practice a muscular relaxation program via a provided CD
Physical activity	Description of potential benefits of physical activity and examples of different forms of physical activity	(i) Perform some form of daily condition-enhancing exercise with a minimal duration of 30 minutes (ii) Perform a provided 5–10-minute exercise program for improved strength, posture, and balance
Diet	Physiology of metabolism; types of foodstuffs; body mass index and abdominal circumference as measures of metabolic health	(i) Increase awareness of personal dietary habits by answering a set of questions (ii) Avoid sugar and other simple carbohydrates.Have meals at regular intervals (iii) Avoid over-eating
Thought patterns	Effects of positive and negative thoughts, respectively; how to identify personal strengths; how to accomplish and maintain changes in thought patterns	(i) Identify personal strengths (ii) Focus on possibilities rather than on difficulties (iii) Sustainably change habits of thought from negative to positive thinking (iv) Improve awareness of realistic limits in daily life and how to implement them
Handling of emotions	How feelings of sadness, anger, envy, and happiness affect thinking and well-being; the roles of feelings and empathy for communication; the concept of emotional intelligence (EQ)	(i) Identify which types of feelings you easily come in contact with and those that are difficult to become aware of (ii) Identify how you react to and are affected by your feelings (iii) Apply various strategies for coping with negative feelings: analyze their causes, verbalize them for those concerned, improve emotional balance via improved relaxation and sleep habits, and avoid unproductive thoughts, e.g. of revenge
Attitudes	How individuals differ in degree of trust in themselves and in others; how trust affects thinking and attitudes	(i) Achieve increased awareness of attitudes of trust/mistrust (one's own and others') (ii) Improve self-reliance and trust in others

### Randomization and study flow

**Figure 1.**
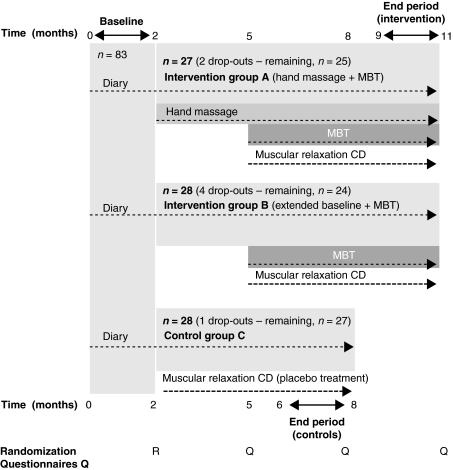
Diagram of the study design with group sizes and drop-outs (MBT = multimodal behavior treatment; Q = questionnaires; R = randomization; CD = compact disc).

The general flow of the study was as follows and is summarized in Figure 1: All participants first recorded their migraine in the diary during a period of 2 months (56 days) as a base-line. Thereafter, the participants were randomized into one of two intervention groups A (*n* = 27; 19 women and 8 men) and B (*n* = 28; 19 women and 9 men) or a control group C (*n* = 28; 20 women and 8 men). The following procedure was used for randomization: a sequence of random numbers was generated in Statistical Package for the Social Sciences 18.0 (SPSS) software, stratified by gender in order to obtain an equal distribution of women and men in the groups. Based on magnitude, these numbers were arranged into three equal-sized groups, which translated into the three study groups. The number sequence thus translated into a unique sequence of group affiliation which corresponded to the chronological order of inclusion. The procedure was performed by an independent researcher, thus the randomization process was blinded to the investigators.

The complete study period was 11 months (308 days) for the intervention groups, and 8 months (224 days) for the control group. Hand massage began immediately after randomization (group A) and was practiced throughout the rest of the study, while participants in intervention group B extended their base-line diary recording for another 3 months (84 days) after randomization. Thereafter, i.e. 5 months (140 days) from the start of the study, MBT was added in both intervention groups (A and B) and lasted for 6 months (168 days). During the first 2 months (56 days) of MBT, the different parts of the program were introduced stepwise at regular intervals via the Internet, gradually building up to the complete program, which the participants were instructed to practice until the end of the study.

The rationale for the study design was to obtain: 1) base-line values unbiased by participants' awareness of which group they would be randomized to; 2) an initial 3-month period of hand massage in one of the intervention groups (group A), while the other intervention group (group B) continued diary registration to enable evaluation of the short-term efficacy of hand massage as the sole form of intervention; 3) an MBT intervention period of sufficient length to learn and practice the program and perceive its effects; and 4) a reasonably long follow-up period of the control group, the assumption being that 11 months, as used in the intervention groups, would be too long for ethical reasons and because the longer period might entail the risk of exhaustion and affect compliance. A CD containing a 15-minute program for muscular relaxation was given to all participants and used as a placebo treatment for the control group (Figure 1) (31). The study design is largely in accordance with the criteria described by Goslin et al. (8).

Aside from communicating via the web tool, participants were also able to make inquiries via e-mail or phone throughout the study. Also, biological sampling (blood and saliva; intended for a separate report) after 5 months (controls) or 5 and 8 months (intervention groups) provided opportunities for 5–10-minute face-to-face communication while the study was underway. The participants in intervention group A also had one face-to-face encounter with one of the researchers when they were taught how to perform hand massage. Thus, all participants experienced a 2-hour initial face-to-face contact upon inclusion, and the total lengths of subsequent face-to-face encounters during the course of the study were estimated to 80 minutes for group A, 20 minutes for group B, and 10 minutes for group C. At the end of the study, after data collection was complete, all participants had a final 5–10-minute study-related face-to-face encounter in connection with biological sampling. At this point in time, participants in the control group were offered access to the MBT program.

### Hand massage

Hand massage was chosen as a possible complementary means of achieving stress reduction and as an easy variant of massage that was not intrusive on participants' integrity. The massage was not intended as a measure of acute alleviation of migraine attacks. The total massage time was 15 minutes per session. The massage was of the ‘Swedish’ type, consisting of: *effleurage* (stroking from the wrist down to the fingers, simultaneously on the palm and on the back of the hand, with use of soft circular stretching strokes), *petrissage* (kneading of the palm), and *friction* (strokes in circles on the fingers) (manual in Swedish provided on request). The collaborator who would perform the massage was chosen by the participant. One of the researchers (K.H.) provided 1 hour's face-to-face training on the massage technique to each participant–collaborator pair, ending with a practical examination. Each pair also received an instructional videotape on how to perform the massage. The participants were asked to perform hand massage at least twice a week during the first 4 weeks and subsequently at least once a week during the remaining 32 weeks of the study period.

### Introduction of questionnaires via the web tool

Two questionnaires were provided via the web tool to all participants for evaluation of depressive symptoms (Montgomery-Åsberg Depression Rating Scale (MADRS-S)) (32,33) and for issues regarding quality of life (PQ23 quality of life questionnaire) (34): at the start of the diary recording and after 5 and 8 months of recording. For participants in the intervention groups, these questionnaires were also provided after 11 months, which was at the end of the intervention (Figure 1). This final evaluation also included a set of four questions, each concerning participants' views on the MBT program and on the hand massage, respectively.

### Questionnaires

The MADRS**-**S is a validated instrument for evaluation of depressive symptoms (32,33,35). It is a self-estimating scale, based on evaluations of the past three days regarding the following nine issues: mood, feeling of unease, sleep, appetite, ability to concentrate, initiative, emotional involvement, pessimism, and zest for life. Seven response options were given for each issue, with ratings running from zero to six. A total score above 12 was defined as a depressive state, with the limits of 20 and 35 defining those with moderate or severe depression, respectively (32,33).

We also included the PQ23 questionnaire (see Appendix). This is a quality of life instrument developed and tested (34) at the Department of Environmental Stress Disorders (CEOS), Uppsala University, Sweden. It is composed of 23 questions, all of which refer to the current situation. We sought a way to group these questions. Therefore, factor analysis was performed on the PQ23 instrument, based on the present answers given at study start, so as to reduce the number of variables and to identify groupings of items. It yielded a four-factor solution with an eigenvalue of >1. The question concerning physical activity was excluded, as it was the only question not included in any of these four factors. The factors were designated as follows: 1: ‘Well-being’ (eigenvalue 8.1; 35.4% of variance explained); 2: ‘Occupational satisfaction’ (2.8; 12.4%); 3: ‘Mood and social satisfaction’ (1.8; 7.8%); 4: ‘Perceived work performance’ (1.4; 5.8%). The internal consistency of scales was tested according to Cronbach's alpha, which yielded values between 0.745 and 0.903.

The participants' opinions about the MBT were obtained by posing four questions. Question 1: ‘How do you rate the clarity of the respective parts of the program?’ Question 2: ‘How do you rate the feasibility of the respective parts of the program?’ Answers were given as a score on a visual analog scale (VAS) scale graded from 0 to 10 (0 = poor; 10 = good). The remaining questions were in an open format: Question 3: ‘Which element(s) of the entire MBT program do you personally consider the most valuable?’ Question 4: ‘Do you have any general suggestions that could improve the program?’

The hand massage was also evaluated using four questions. Answers were given as a VAS score on a scale from 0 to 10: Question 1: ‘How clear was the information given at the training session on how to perform the hand massage?’ (0 = poor; 10 = good). Question 2: ‘How clear were the instructions given on the videotape on how to perform the hand massage?’ (0 = poor; 10 = good). Question 3: ‘What were your emotional impressions of the hand massage?’ (0 = negative; 10 = positive). Question 4: ‘Did the hand massage have an effect on your migraine?’ (0 = negative; 10 = positive).

### Analyses of data

#### Multivariate analyses

SPSS's generalized linear model with a binary response variable (50% or more reduction of migraine frequency or not) and a logit link was used. The model was set up with the main factors *Intervention* and *Gender*. *Base-line migraine frequency* and *Change in physical activity* were used as covariates. The interaction effects *gender × intervention*, *gender × base-line migraine frequency* and *gender × change in physical activity* were also included in the model. The parameter estimates from the model were presented as odds ratios (OR) and 95% confidence intervals (95% CI). The pairwise comparisons of estimated marginal means were based on the original scale of the dependent variable, i.e. probability of reducing one's migraine frequency level with 50% or more. The significance levels were adjusted for multiple testing with sequential SIDAK correction.

#### Univariate intergroup comparisons: three-group comparisons

Dichotomous parameters differences in proportions were analyzed using the chi-square test (gender, marital status, children living at home, presence of tension-type headache, migraine with or without aura, and depression). Group differences regarding continuous parameters were examined using one-way ANOVA with Tukey *post-hoc* test (age, body mass index (BMI), migraine frequency, and years of migraine). Differences in proportions of subcategories between groups were analyzed using the Kruskal–Wallis test (income, education, employment). Two-way analysis of variance (ANOVA), including interaction effect analysis, was used for evaluation of the repeated measures of the MADRS-S and the PQ23 questionnaires. Initial values from these questionnaires, obtained at study start, were used as the reference for all comparisons.

#### Univariate intergroup comparisons: two-group comparison

The Mann–Whitney *U*-test was used in the early evaluation of the efficacy of the hand massage program. Group B constituted the control group, and the changes in migraine frequency between days 1–28 and 57–84 of the hand massage group A and of group B were compared.

### Factor analysis

Based on participants' initial answers to the PQ23 questionnaire a factor analysis was performed using principal components analysis and Varimax rotation (36). The number of latent factors was determined using an eigenvalue above 1. Cronbach's alpha was used as a measure of the reliability of each component.

### Power analysis

A prospective power analysis had been conducted, based on the calculations presented in another, similar MBT study on chronic migraine (9), indicating a need for 36 participants in each study group. All analyses were performed with SPSS 18.0 software. Significance level was uniformly set at 0.05, two-tailed test.

### Qualitative analysis

A qualitative analysis, inspired by the method of manifest content analysis (37), was performed on answers to the open-ended question regarding participants' opinions as to which topic(s) of the behavioral treatment program had been most valuable. In this analysis, performed manually by the authors, representative parts of the written answers were first chosen and brought together in meaningful units. Subsequently, these were condensed with the intention of keeping the content intact but in a condensed format. The condensed text was then coded and sorted into categories, constituting the main content of the answers.

## Results

### Demographics and migraine characteristics

Background socio-economic and migraine characteristics, including the migraine frequency during the base-line period as recorded in the diary, are shown in Table II; these revealed that the three randomized study groups differed in annual income and degree of employment at study start, but were otherwise strikingly similar.

**Table II.** Demographic profiles and migraine characteristics of participants in intervention groups A and B and in control group C.

	Group A (hand massage + MBT) *n* = 25	Group B (extended base-line + MBT) *n* = 24	Group C (controls) *n* = 27	Chi-square/F values	*P* value
Gender (%)^a^					
Women	68.0	66.7	70.4	χ^2^;0.08	0.956^c^
Men	32.0	33.3	29.6		
Age (years)					
Mean	49.4	44.8	49.0	F; 1.36	0.291^d^
95% CI	44.4–54.3	40.1–49.5	45.4–52.5		
Range	22–65	23–61	27–65		
Body mass index					
Mean	24.9	25.2	25.5	F; 0.15	0.855^d^
95% CI	23.5–26.3	23.7–26.6	23.7–27.4		
Range	19.0–35.3	18.6–30.7	19.5–38.6		
Income per year (%)^a^					
>40,000 €	20.0	8.3	11.1		
25,000–40,000 €	40.0	25.0	37.0	χ^2^; 6.04	0.049^e^
10,000–25,000 €	36.0	41.7	51.9		
<10,000 €	4.0	25.0	0		
Education (%)^a^					
College/university/post-graduate studies	68.0	66.7	48.1		
Upper secondary school	24.0	25.0	40.7	χ^2^; 2.39	0.303^e^
Nine-year compulsory school/elementary school	8.0	8.3	11.1		
Employment (%)^a^					
Full-time	56.0	58.3	85.2		
Part-time	28.0	20.8	11.1	χ^2^; 6.26	0.044^e^
Pension/unemployed	16.0	20.8	3.7		
Marital status and children (%)^a^					
Married/partner	72.0	75.0	81.5	χ^2^; 0.68	0.712^c^
Single	28.0	25.0	18.5		
Married/partner and children living at home	36.0	50.0	30.6	χ^2^; 2.31	0.315^c^
Single parent with children living at home	12.0	–	11.1	χ^2^; 3.02	0.221^c^
Migraine frequency during base-line recording (56 days)					
Mean	10.1	13.9	10.0	F; 2.05	0.182^d^
95% CI	7.2–12.9	10.2–17.6	7.2–12.9		
Range	1–27	1–33	2–33		
Tension-type headache	32.0	41.7	37	χ^2^; 0.49	0.782^c^
Aura^b^ (%)^a^	48.0	29.2	51.9	χ^2^; 2.97	0.225^c^
Years of migraine					
Mean(SD)	23.1	22.2	24.3	F; 0.18	0.822^d^
95% CI	17.7–28.4	17.0–27.5	19.9–28.7		
Range	7–49	7–46	7–46		

^a^Percent of participants in the group.

^b^Scintillation, numbness, and difficulties in speaking.

^c^Chi-square analysis was used for statistical comparisons.

^d^One-way ANOVA with Tukey *post-hoc* test analysis was used for statistical comparisons.

^e^Kruskal–Wallis Test analysis was used for statistical comparisons.

SD = standard deviation; CI = confidence interval for mean.

### MBT outcome: group-specific changes in migraine frequencies

**Figure 2.**
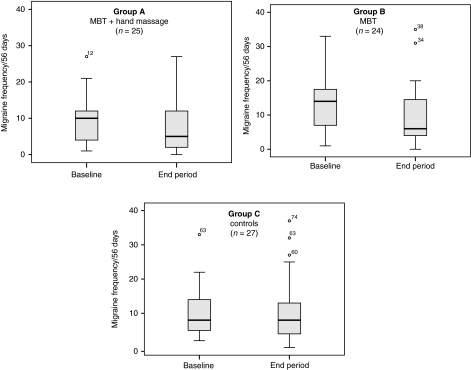
Change in migraine frequency between base-line and end period registrations. Box plot diagrams show median value, interquartile range, and full range of number of days with migraine during the first (base-line period) and the last (end period) 56 days of the study for the respective study groups. Outliers are represented by circles and personal codes.

**Figure 3.**
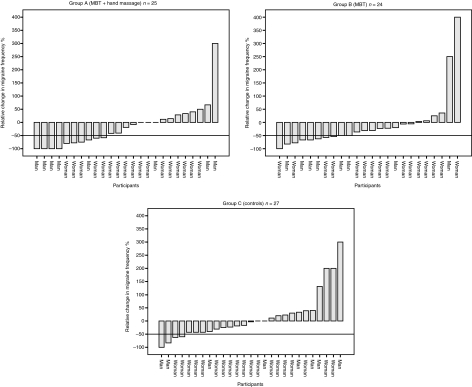
Relative change in migraine frequency between base-line and end period registrations. Individual changes, measured as the percentage decrease or increase of migraine frequency during end period registration compared to the base-line period, are shown in order of magnitude for the respective study groups. Horizontal lines define our main outcome measure: 50%, or more, decreased migraine frequency.

Figure 2 shows base-line and end period distributions of migraine frequencies for the three study groups. Relative change in migraine frequency at the individual level is shown for the three study groups in Figure 3. In the intervention groups A, B, and C, respectively, 40%, 42%, and 15% of participants reported 50% or more improvement in headache frequency at the end period of the study (Figure 3).

### Multivariate intergroup comparisons

#### Main factors

Multiple logistic regression revealed that the intervention itself was the most interesting factor, with a significant effect (*P =* 0.039) (Table III). Pairwise comparisons revealed that both treatment groups (A and B) had significantly higher proportions of participants who had reduced their migraine frequency by 50% or more compared to the control group (*P* = 0.022 and *P =* 0.031, respectively); no significant difference in reduction of migraine was seen between the two treatment groups A and B (Table III). No significant overall effect of gender was seen (Table III).

**Table III.** Main outcome and interactions. A multivariate analysis was performed based on the main outcome variable, a 50% or greater decrease in migraine frequency. Tested main factors were: study intervention and gender. Base-line migraine frequency and change in physical activity between the base-line period and the end period were tested as covariates. Interactions between gender and these other variables were also tested.

Model effect (Type III)	*P* value			
Main factors				
Intervention (study groups)	0.039			
Pairwise difference^a^		mean difference	95% CI	
A versus C	0.019	0.56	0.07	1.06
B versus C	0.037	0.41	0.02	0.80
A versus B	0.463	0.15	−0.26	0.57
Gender	0.891			
Covariates				
Base-line migraine frequency	0.490			
Change in physical activity	0.119			
Gender interactions				
Gender × intervention (study groups)	0.505			
Gender × base-line migraine frequency	0.093			
Gender × change in physical activity	0.077			
Interaction effects		OR	95% CI	
Women × base-line migraine frequency versus men × base-line migraine frequency		0.79	0.60	1.04
Women × change in physical activity versus men × change in physical activity		1.19	0.98	1.44

^a^Adjusted for multiple tests by sequential SIDAK.

#### Covariates

The effect of the covariate *base-line period migraine frequency* was not significant, which shows that results were not affected by such differences between the groups. The effect of change in physical activity comparing base-line period to end period was not significant either.

### Gender interactions

The interaction analyses of gender revealed tendencies indicating that women with a high level of migraine at base-line might have less chance for improvement than men. On the other hand women who increased their physical activity during the study might have a somewhat better chance of improvement than men. However, none of these gender effects were significant (*P =* 0.093 and *P =* 0.077) (Table III).

### Hand massage outcome

**Figure 4.**
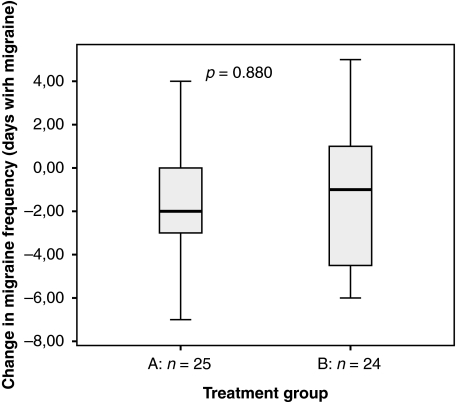
Early evaluation of hand massage efficacy in alleviating migraine. The changes in number of days with migraine between the first and the last 4 weeks of an initial 3-month period of hand massage (study group A) versus no treatment (study group B) are shown separately by use of box plot diagrams showing median value, interquartile range, and full range of these changes. Mann–Whitney *U*-test was used for statistical comparison between the groups.

**Figure 5.**
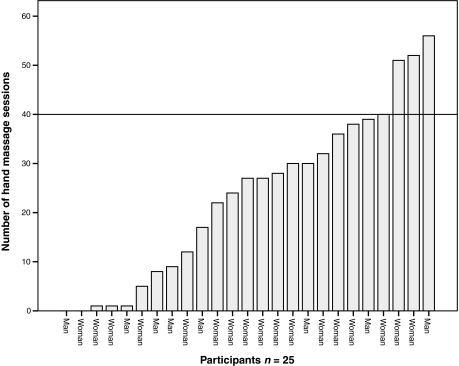
Self-reported compliance of hand massage. Forty sessions were recommended as indicated by the line.

Analysis of the short-term efficacy at 3 months of hand massage revealed no significant effect on migraine frequency compared to that of group B (*P* = 0.880) (Figure 4). As mentioned above and shown in Table III, no effect by adding hand massage was seen on our main outcome measure, 50% or more reduction in migraine frequency, at the end period of the intervention (*P =* 0.803; A versus B). Self-reported compliance during the whole period of hand massage is shown in Figure 5; average compliance decreased from 81% to 44% of the recommended number of sessions after introduction of the MBT program, which is equal to 1.1 sessions/week during the first 12 weeks and to 0.44 sessions/week during the last 24 weeks of hand massage (*n* = 25).

### Questionnaire results

#### MADRS-S depression scale

At study start, the proportion of participants with clinical signs of depression according to the MADRS-S depression inventory (score >12) was 16.0% in intervention group A (*n* = 4; score range 13–29), 38.0% in intervention group B (*n* = 9; score range 13–21), and 18.5% in the control group (*n* = 5; score range 13–14). These proportions of depressed participants did not differ significantly between the groups (*P =* 0.153; chi-square). No significant differences were found in average MADRS-S scores when comparing the base-line starting-point with multiple time points throughout the study period in any of the study groups (Table IV), and analyses of interaction effects did not indicate any significant differences in change of depression scores over time between the groups (Table IV).

**Table IV.** Average scores and standard deviations for depression (MADRS-S scale). Comparisons according to the two-way repeated measures analyses of variance (ANOVA), including analyses of interaction effects, between intervention group A, intervention group B, and control group C.

	At start mean (SD)	After 5 months mean (SD)	*P* value (5 mo versus start)	After 8 months mean (SD)	*P* value (8 mo versus start)	After 11 months mean (SD)	*P* value (11 mo versus start)
MADRS-S:							
Intervention group A (*n* = 23^a^)	7.8 (6.6)	8.1 (7.3)	0.772	5.8 (6.1)	0.026	6.9 (7.1)	0.311
Intervention group B (*n* = 23^a^)	9.3 (6.4)	8.9 (6.5)	0.621	8.9 (6.9)	0.663	8.7 (7.5)	0.579
Control group C (*n* = 25^a^)	6.6 (4.6)	6.8 (5.2)	0.851	5.8 (7.7)	0.489	–	–
Interaction effect(group × time)			0.821		0.559		

^a^Missing data from two participants of intervention group A, from one participant in intervention group B, and from two participants of the control group C.

#### PQ23 quality of life scale

The only statistically significant and stable improvement observed over time in the four factors of the PQ23 inventory was ‘Perceived work performance’ in intervention group A, which was improved compared to the base-line starting-point at all follow-up time points (Table V). Concerning the other three factors of this inventory (i.e. well-being, occupational satisfaction, and mood and social satisfaction), none of the study groups displayed significant differences between study start and any of the follow-up time points throughout the study period (Table V). Analyses of interaction effects yielded significant values only for ‘Perceived work performance’ at the 8-month time point, showing that while group C remained at a constant level throughout the study, both groups A and B showed lower values at 8 months for this factor, compared with base-line (Table V).

**Table V.** Average scores and standard deviations for quality of life (PQ23 scale). Comparisons according to the two-way repeated measures analyses of variance (ANOVA), including analyses of interaction effects, between interventions group A, intervention group B, and control group C.

	At start mean (SD)	After 5 months mean (SD)	*P* value (5 mo versus start)	After 8 months mean (SD)	*P* value (8 mo versus start)	After 11 months mean (SD)	*P* value (11mo versus start)
PQ23:							
Well-being							
Intervention group A (*n* = 23^a^)	36.7 (15.4)	37.1 (19.7)	0.917	35.3 (23.4)	0.747	34.8 (24.4)	0.658
Intervention group B (*n* = 23^a^)	39.2 (18.6)	40.1 (16.7)	0.730	35.0 (20.1)	0.304	43.3 (21.5)	0.336
Control group C (*n* = 25^a^)	35.7 (17.6)	38.6 (17.6)	0.377	32.0 (21.3)	0.340	–	–
Interaction effect (group × time)			0.812		0.870		
Occupational satisfaction							
Intervention group A (*n* = 23^a^)	32.0 (24.4)	34.3 (23.6)	0.609	33.4 (20.9)	0.773	31.5 (24.7)	0.926
Intervention group B (*n* = 23^a^)	40.8 (25.9)	38.5 (18.4)	0.609	35.1 (21.1)	0.042	38.4 (22.6)	0.644
Control group C (*n* = 25^a^)	27.3 (17.9)	32.7 (22.1)	0.100	29.8 (20.5)	0.409	–	–
Interaction effect (group × time)			0.404		0.229		
Home situation/mood							
Intervention group A (*n* = 23^a^)	27.1 (20.8)	30.4 (22.2)	0.543	26.0 (21.7)	0.836	27.6 (22.0)	0.930
Intervention group B (*n* = 23^a^)	32.0 (19.6)	28.9 (15.4)	0.246	27.8 (20.6)	0.204	31.4 (20.1)	0.852
Control group C (*n* = 25^a^)	25.1 (18.2)	25.0 (14.1)	0.989	23.4 (16.7)	0.537	–	–
Interaction effect (group × time)			0.526		0.838		
Perceived work performance							
Intervention group A (*n* = 23^a^)	70.5 (21.2)	56.0 (20.6)	0.022	50.9 (18.2)	0.001	51.4 (22.5)	0.001
Intervention group B (*n* = 23^a^)	60.1 (20.5)	58.2 (21.6)	0.681	50.5 (24.9)	0.078	59.2 (18.8)	0.802
Control group C (*n* = 25^a^)	63.2 (15.9)	64.1 (19.1)	0.839	62.5 (19.2)	0.880	–	–
Interaction effect (group × time)			0.072		0.026		

^a^Missing data from two participants of intervention group A, from one participant in intervention group B, and from two participants of the control group C.

#### Participants' evaluation of the MBT program

After maximally two reminders (letter followed by telephone call), a total of 42 of the 49 participants in the MBT program (24/25 from group A and 18/24 from group B) responded to the questions with VAS answers about the MBT program. The clarity of the program was rated on a VAS scale (0 = poor, 10 = good); the mean was 8.0 and the range 4.6–10. The feasibility of the program had a mean score of 5.9, with a 0.6–10 range. Thirty-eight of the responders also gave written answers to question 3: 95% of the answers rated the cognitive aspects of the program dealing with emotions, thoughts, and attitudes as the most rewarding parts of the MBT program. Guided by the principles of manifest content analysis, these answers were divided into three categories: ‘Perspectives on my life situation’, ‘Increased knowledge’, and ‘Possibilities for change’. Quotations from representative answers are presented in Table VI. Concerning answers regarding possible improvements of the program (question 4), the majority of participants reported that, overall, they were satisfied with the program's design. Proposed improvements were: combining the web-administered program with other forms of follow-up such as group meetings or opportunities to chat over the Internet with other participants, or private follow-ups with a professional tutor. More literature references were also requested.

**Table VI.** Selected quotations of answers to the question ‘Which element(s) in the entire MBT program do you personally consider the most valuable?’ grouped according to the result of a content analysis.

Perspectives on my life situation:
‘Increased awareness of what causes negative stress in life’
‘Better understanding of the connection between a stressful period and a migraine attack’
‘To understand that my own thoughts and emotions are just as important as those of other people’
‘Knowledge about patterns of thought was the most fruitful part of the program and I increased my self-awareness, allowing me to understand myself more easily’
‘I have learned to put into words what I feel—you can’t blame others for the feelings they arouse in you, but you can make clear to the person what you feel’
‘I have realized and accepted that emotions are an important part of life, which I previously had rejected. I have opened up a bit and learned how quickly feelings of happiness or of discomfort come to you. This insight into how feelings come to you has been rewarding, and increased awareness of my own feelings has sometimes been helpful in deciding how to react in a specific situation’
Increased knowledge:
‘Knowledge of brain functions has been valuable—it has deepened my understanding of personal experiences’
‘The CD on muscular relaxation was valuable. It gave an opportunity to experience the difference between being tense and being relaxed’
‘Increased awareness of the importance of a balanced diet’
‘Increased awareness of the importance of food habits for migraine’
‘Interesting that thoughts lead to physical and emotional experiences’
‘I have come to understand a little more about my own thought patterns and how they can influence my health—I knew this already but it was good to get it in writing’
‘A great awakening regarding the importance of my way of thinking and its impact on my mode of life’
Possibilities for change:
‘To realize the importance of planning your time in order to avoid stress’
‘I have started to practice physical exercise and I have found that it diverts my mind from thinking about problems’
‘I have radically changed my eating habits’
‘I really appreciated reading about the different intelligences. I have started focusing more on possibilities and I am trying to abandon bad thoughts about myself’
‘I realized that I was focusing on my own imperfections more than I thought—Now I say no to things and avoid just struggling on without first reflecting’
‘I have difficulties with changing my thought patterns—I have to work a lot on this’
‘I have allowed myself to have positive feelings even though this has resulted in occasional backlashes’
‘To learn how to change my thoughts in order to feel less tense when experiencing unpleasant situations’
‘You can’t change other people—first you must change yourself’

#### Participants' evaluation of the hand massage

This showed the following: clarity of the educational session: mean VAS score 9.5; range 8.5–10 (0 = poor, 10 = good), clarity of videotape instructions: mean VAS score 9.5; range 8.5–10 (0 = poor, 10 = good), emotional impact of the massage: mean score 9.0; range 6.2–10 (0 = negative; 10 = positive), subjective evaluation of the ability of the massage to alleviate migraine symptoms: mean score 6.0; range 4.7–8.8 (0 = negative; 10 = positive). Complete answers were given by 18 of the 25 participants in group A, corresponding to a response rate of 72%.

### Migraine medication

Data on acute migraine medication were recorded and will be reported separately. Preliminary data indicate that such drug use decreased in the intervention groups but not in the control group C. Fourteen participants were on continuous preventive medication throughout the study, and five participants underwent a change in preventive medication during the course of the study, almost evenly distributed between the groups.

### Attrition

Seven of the initial study participants dropped out during the study. The gender and group affiliation of the drop-outs were as follows: two women in group A, three women and one man in group B, and one woman in the control group C (Figure 1). The reasons were: family situation (two women and one man), divorce, cancer diagnosis, moving from the region, and for one participant for an unknown reason. Data on these individuals were not included in the study. We analyzed drop-outs versus the remaining participants for differences in demographics and background migraine characteristics and found no statistical evidence for any differences (data not shown). Two participants in the hand massage intervention group (group A) were not able to find a hand massage collaborator, and three others never carried out their hand massage. An intention-to-treat approach was used for evaluating the effects of hand massage, thus including the data of these five participants despite their non-compliance.

## Discussion

### Main findings regarding multimodal behavioral treatment

The main objective of the present study was to develop and evaluate an Internet-administered multimodal behavioral treatment (MBT) program for migraine. Our web-based platform appears to be unique in specifically addressing this disorder, and we consider the design of our MBT program promising, as our intention to obtain decreased suffering from migraine was achieved. Based on our measure of a 50% or greater reduction in headache frequency, we found a significantly higher rate of improvement among participants of the MBT than in controls. This relative measure of migraine improvement is a preferred outcome measure, as suggested by Goslin et al.(8). When looking at migraine improvement in absolute figures, participants of the study groups receiving MBT showed 23% and 29% average improvement in migraine frequency, respectively, compared to none in the control group. Considering the high prevalence of migraine and the high accessibility of Internet-administered behavioral treatment, we regard the present result to be of health economical significance.

The effects of our MBT intervention were studied as a whole, with limited ambitions to sort out the relative contributions of individual constituents of the program. However, we did perform a multivariate analysis in which the potential effect of increased physical activity was analyzed. We were not able to show an overall effect, but a tendency for an interaction with gender hinted at the possibility that specifically women's migraine might profit from increased physical activity. When analyzing gender in our multivariate model we found no significant contribution to the obtained effect by this variable, arguing that female and male migraineurs have similar benefit from our program.

One further result, a low attrition rate, was a considerable advancement compared to other Internet-based studies for headache treatment, which have shown attrition rates in the 32%–56% range (27–29). Our rate of 8.4% conforms to the recommended less than 20% attrition rate for studies on behavioral therapy for migraine, as suggested in an evidence-based Cochrane protocol for such studies (38).

### Hand massage

We were not able to demonstrate any migraine-alleviating effect of massage in the present study. It is noteworthy that compliance with the hand massage program during the initial 3 months, prior to MBT, was high, with a 1.1 sessions per week rate (recommended rate: 16 session/12 weeks), but decreased after onset of the MBT program resulting in an average practice rate of less than once a fortnight (recommended rate: once weekly). This low massage frequency during the latter part of the study definitely constitutes a power problem of the hand massage study. The fact that not even during the first 3 months could an effect be seen argues more strongly against any greater effect of massage on migraine, despite our sincere efforts to elucidate this. Nonetheless, it is noteworthy that many participants appreciated highly the positive emotional impact of the massage and that our format appeared fully feasible for a substantial number of participants.

### Questionnaire evaluation

The written evaluation of the MBT program clearly showed that participants appreciated its content, witnessing that it was helpful in achieving improved knowledge on the importance of stress and that they had been able to integrate its cognitive aspects into their daily lives.

Regarding depressive symptoms we saw no evidence of a general effect in the present study. It is well known that the prevalence of depression is higher in migraine populations (39–41), and, judging from the MADRS-S scores, participants in the present study did show a higher prevalence of depression than that found in the general population (42). At the same time, the number of participants in each group with symptoms of depression was too low to make any valid statistical analyses.

The PQ23 quality of life instrument used here showed some effects in the ‘Perceived work performance’ factor, and this was seen exclusively for participants in the hand massage group. We consider that this finding could have been biased by the low base-line value for the mentioned factor, specifically in this treatment group. We therefore conclude that no reliable improvement in quality of life was detected.

### Design, representativeness, and limitations

The design of the present study differed from other similar headache studies in that it included also participants with more moderate migraine frequency by using a limit of at least two monthly attacks, compared to at least four (10,27–29), at least eight (3) attacks/month, or chronic (9) symptoms.Because of this, and due to the character of our MBT program, longer base-line and treatment periods than used in these mentioned studies were deemed necessary. The feasibility, shown here, of using such long periods may be of interest for future studies. It is furthermore noteworthy that our design accords quite well with the criteria for optimal behavioral intervention studies in migraine, as formulated in the meta-analysis by Goslin et al. (8): use of headache frequency as the outcome measure—as opposed to headache index; use of a prospective base-line period and an end recording period of at least 4 weeks each (8 weeks each in the present study); a treatment period of at least 3 months (6 months' MBT treatment and 9 months' hand massage in the present study), and use of 50% or more reduction in headache frequency as the criterion for individual response. Our study also complied with the more recent recommendations of the above-mentioned Cochrane protocol on migraine (38), with use of a daily headache diary and an attrition rate not exceeding 20%.

We believe that the low attrition rate in our study was boosted by the compulsory format of the diary as well as the face-to-face contacts, which were not used in the previously mentioned Internet-based headache studies (27–29). We believe that the thorough initial face-to-face contact with all participants at the time of inclusion in the study was psychologically particularly important for achieving our low attrition rate. Our control group showed the lowest attrition rate. One possible explanation for this could be that the MBT program is fairly demanding as regards both personal engagement and time needed for its different activities. The balance between potential benefits and how much engagement the person is willing and able to invest is important to take into account when designing a program such as this. We experienced a considerably smaller attrition problem than reported in six of eight studies in a review on Internet-based behavioral treatment programs for a variety of health problems (23). Interestingly, only one of these eight studies used a face-to-face introduction to the program, and this was one of the two studies with a low attrition similar to ours (43), thus giving further support to our presumption that an initial personal contact is important for subsequent attendance to Internet-administered treatment. When compared with other headache studies, our attrition rate is more comparable with that of a somewhat similar face-to-face study on migraine by Lemstra et al. (3.8% attrition rate; *n* = 80) (9) than with the Internet-based headache studies we have identified (27–29). Similar to our study, an MBT approach was used in the study by Lemstra et al., including issues of diet, physical activity, cognitive change, and massage. However, the study by Lemstra et al. differed by including only participants with chronic migraine, who were also younger on average, and by using a 6-week face-to-face group session design.

In the present study, we chose not to interfere with any on-going pharmacological treatment, and when checking for any change in preventive medication only very few changed their medication. We do not believe this had any impact on the outcome.

It is likely that a larger sample size with more power would have yielded clearer results, although the present size was evidently sufficient for determining that our format for MBT has an effect. Although we would have liked to include more participants, our sample size compares well with those of recent (3,9,10,27–29) and older studies (before 1999: reviewed in (8)) on behavioral treatment of migraine. Furthermore, gender aspects are of importance in a disease such as migraine (44–47),and gender-based analyses may generate new knowledge. However, despite our extra efforts to recruit men, we consider their number suboptimal for this purpose.

### Concluding remarks and prospects for the future

We conclude that our MBT program, in its present format, seems to improve migraine, presumably via increasing awareness of, and adjustments to, healthier life-style patterns. The results, showing both efficacy and a low attrition rate, speak in favor of using the Internet for behavioral treatment of migraine. We therefore believe our program has the potential of becoming a valuable and effective tool at a probably limited cost in the treatment of migraine. However, further evaluation of the program is needed for better understanding of the individual contributions of its components, its long-term effects, and its full potential for improvement.

## NOTICE OF CORRECTION

[ePub ahead of print] 20 April 2011, DOI: 10.3109/03009734.2011.575963. The Early Online version of this article published ahead of print on 20 April 2011 contained an error on page 184, line 786 and on page 185 where reference 47 was missing. This has now been corrected.

## Appendix

The PQ23 instrument developed at the Department of Environmental Stress Disorders (CEOS), Uppsala University, Uppsala, Sweden.

**Table:**

It consists of the following 23 items:	Response options were given on a VAS scale for each item, graded from 0 to 100	

	0	100

1. Health status right now?^1^	excellent	poor
2. Feeling of great strain right now?^1^	do not agree	agree totally
3. Day-time physical exhaustion?^1^	rarely	very often
4. Day-time mental tiredness?^1^	rarely	very often
5. Frequency of physical exercise?^a^	often	never
6. Fatigue right now?^1^	never	daily
7. Depression/sadness right now?^3^	never	daily
8. Restlessness right now?^3^	never	daily
9. Satisfaction with working conditions?^2^	excellent	poor
10. Own influence on working conditions?^2^	agree totally	do not agree at all
11. Stimulation at work?^2^	excellent	poor
12. Positive feedback for good performance at work?^2^	often	never
13. General feeling right now?^1^	excellent	poor
14. Quality of sleep right now?^1^	excellent	poor
15. Ability to concentrate right now?^1^	excellent	poor
16. Stress level right now?^1^	not stressed	very stressed
17. Life control right now?^3^	agree totally	do not agree at all
18. Energy level right now?^1^	excellent	poor
19. Intensity of work right now?^4^	low	high
20. Efficacy in work performance right now?^4^	excellent	poor
21. Satisfaction with home conditions right now?^3^	satisfied	dissatisfied
22. Satisfaction with social life right now?^3^	satisfied	dissatisfied
23. Degree of overexcitement^4^	low	high

1–4 refer to factor analyses grouping, see Material and methods.

^1^Well-being,

^2^Occupational satisfaction,

^3^Mood and social satisfaction,

^4^Perceived work performance.

^a^Excluded.
